# Production Optimization, Structural Analysis, and Prebiotic- and Anti-Inflammatory Effects of Gluco-Oligosaccharides Produced by *Leuconostoc lactis* SBC001

**DOI:** 10.3390/microorganisms9010200

**Published:** 2021-01-19

**Authors:** Minhui Kim, Jae-Kweon Jang, Young-Seo Park

**Affiliations:** 1 Department of Food Science and Biotechnology, Gachon University, Gyeonggi-do 13120, Korea; vanille28410@gmail.com; 2 Food Nutrition Major, School of Food, Chungkang College of Cultural Industries, Icheon 17390, Korea; jkjang@chungkang.ac.kr

**Keywords:** lactic acid bacteria, *Leuconostoc lactis*, oligosaccharides, prebiotic activity, glucansucrase, anti-inflammatory activity

## Abstract

*Leuconostoc lactis* SBC001, isolated from chive, produces glucansucrase and synthesizes oligosaccharides through its enzymatic activity. This study was conducted to optimize oligosaccharide production using response surface methodology, analyze the structure of purified oligosaccharides, and investigate the prebiotic effect on 24 bacterial and yeast strains and the anti-inflammatory activity using RAW 264.7 macrophage cells. The optimal conditions for oligosaccharide production were a culture temperature of 30 °C and sucrose and maltose concentrations of 9.6% and 7.4%, respectively. Based on ^1^H-NMR spectroscopic study, the oligosaccharides were identified as gluco-oligosaccharides that consisted of 23.63% α-1,4 glycosidic linkages and 76.37% α-1,6 glycosidic linkages with an average molecular weight of 1137 Da. The oligosaccharides promoted the growth of bacterial and yeast strains, including *Lactobacillus plantarum*, *L. paracasei*, *L. johnsonii*, *Leuconostoc mesenteroides*, *L. rhamnosus*, and *Saccharomyces cerevisiae*. When lipopolysaccharide-stimulated RAW 264.7 cells were treated with the oligosaccharides, the production of nitric oxide was decreased; the expression of inducible nitric oxide synthase, tumor necrosis factor-α, interleukin (IL)-1β, IL-6, and IL-10 was suppressed; and the nuclear factor-kappa B signaling pathway was inhibited. In conclusion, the gluco-oligosaccharides obtained from *Leu. lactis* SBC001 exhibited a prebiotic effect on six bacterial and yeast strains and anti-inflammatory activity in RAW 264.7 macrophage cells.

## 1. Introduction

The metabolic activities and microbial composition of intestinal microbiota are associated with a variety of diseases [1]. The intestinal microbiota maintain the normal function of the human digestive system and establish host resistance to pathogens in the intestinal tract [2]. Probiotics are defined as “a live microbial supplement that beneficially affects the host animal by improving its intestinal microbial balance” [3]. Probiotics have beneficial effects on the host such as the suppression of infectious diseases and promotion of digestion and the intestinal flora balance [1,4]. Probiotics include lactic acid bacteria (LAB) such as *Lactobacillus* sp. and bifidobacteria as well as some yeast species such as *Saccharomyces cerevisiae* var. *boulardii* [3].

Some probiotic strains produce oligosaccharides that cannot be digested by human digestive enzymes. These non-digestible oligosaccharides can be used as prebiotics that stimulate the growth of probiotic strains [5]. Oligosaccharides are low molecular weight carbohydrates with a degree of polymerization (DP) from 3 to 10, and prebiotic oligosaccharides consist of inulin, fructo-oligosaccharides (FOSs), galacto-oligosaccharides, isomalto-oligosaccharides (IMOs), mannan-oligosaccharides, soybean oligosaccharides, and lactulose [6,7]. Oligosaccharides, as prebiotics, improve the growth of lactobacilli and bifidobacteria and act as precursors of the production of short-chain fatty acids (SCFAs), and these SCFAs, such as acetate, lactate, propionate, and butyrate, maintain the homeostasis of the host, including the enhancement of immune function and improvement of the bioavailability of minerals [8,9,10,11,12,13,14].

Typically, oligosaccharide-producing LAB include the genera *Leuconostoc*, *Weissella*, *Streptococcus*, *Lactobacillus*, and *Oenococcuss*; these LABs synthesize oligosaccharides by the action of glucansucrase (EC 2.4.1.5), a type of glycosyltransferase, using sucrose as substrate. In addition to glucansucrase, mutansucrase (EC 2.4.1.5), alternansucrase (EC 2.4.1.140), and levansucrase (EC 2.4.1.10) synthesize oligosaccharides with different structures [15,16,17].

*Leuconostoc* sp. is a heterofermentative LAB that produces lactic acid as well as diacetyl, CO_2_, acetoin, acetate, ethanol, and 2, 3-butylenglycol [18]. *Leuconostoc* sp. is generally found in raw milk and cheese and used as a starter in the dairy industry [19]. Although numerous studies have investigated the oligosaccharides produced by *Leu. mesenteroides*, only a few studies have been conducted on the oligosaccharides produced by *Leu. lactis* [16,17,20,21,22].

*Leu. lactis* SBC001, which is isolated from chives, produces oligosaccharides in good yield [23]. This strain exhibits glucansucrase activity and produces oligosaccharides using sucrose and maltose as a donor molecule and receptor molecule, respectively [23].

In the present study, we determined the optimum conditions of oligosaccharides production by *Leu. lactis* SBC001 using response surface methodology (RSM) and also evaluated their structure. We also investigated the prebiotic effect of oligosaccharides obtained from *Leu. lactis* SBC001 on the growth of microorganisms and the anti-inflammatory effect of oligosaccharides in RAW 264.7 macrophage cells in vitro using real-time quantitative polymerase chain reaction (RT-qPCR) and Western blotting analysis.

## 2. Materials and Methods

### 2.1. Lactobacillus Strains and Growth Conditions

The oligosaccharide-producing strain *Leu. lactis* SBC001 was originally isolated from chives [23]. This strain was maintained on lactobacilli de Man Rogosa Sharpe (MRS; BD, Franklin Lakes, NJ, USA), which consisted of 2 g/L dipotassium hydrogen phosphate, 20 g/L glucose, 0.2 g/L magnesium sulfate heptahydrate, 0.05 g/L manganous sulfate tetrahydrate, 8 g/L meat extract, 10 g/L peptone, 5 g/L sodium acetate trihydrate, 2 g/L triammonium citrate, and 4 g/L yeast extract, at 37 °C for 24 h.

### 2.2. Optimization of Oligosaccharide Production from Leu. lactis SBC001 Using Experimental Design

Oligosaccharides were produced by *Leu. lactis* SBC001 using LM broth that consisted of 2 g/L peptone, 4 g/L yeast extract, 0.02 g/L sodium chloride, 2 g/L potassium phosphate, 0.4 g/L magnesium sulfate, 0.02 g/L ferrous sulfate, 0.02 g manganese (II) sulfate, 0.03 g/L calcium chloride, and appropriate amounts of sucrose and maltose. RSM was used for the optimization of oligosaccharide production conditions using Minitab 17.2.1 (Minitab Pty Ltd., Sydney, Australia). The initial pH, fermentation temperature, and sucrose concentration were chosen for a central composite design. In this study, the experimental design consisted of the following independent variables: the initial pH (4.0, 5.0, 6.0, 7.0, and 8.0), sucrose concentration (0.1, 0.2, 0.3, 0.4, and 0.5 M), and culture temperature (25, 29, 33, 37, and 41 °C) at five levels (−2, −1, 0, +1, and +2), leading to 20 runs (Tables S1 and S2).

### 2.3. Purification of Oligosaccharides

Oligosaccharides produced by *Leu. lactis* SBC001 were purified as described in a previous study [17]. The culture supernatant was concentrated 10-fold at 60 °C using a rotary evaporator (SB-1200, EYELA, Miyagi, Japan), and the concentrate was loaded onto Bio-gel P2 resin (Bio-Rad Laboratories, Inc., Hercules, CA, USA) packed in a glass Econo-Column (1.5 × 120 cm, Bio-Rad Laboratories, Inc.). The elutes were collected using a fraction collector (Gilson Inc., Middleton, WI, USA) with a fraction volume of 5 mL per tube with a flow rate of 0.5 mL/min. The fractions were analyzed by thin-layer chromatography (TLC), and the oligosaccharide fractions were pooled and freeze-dried using a freeze dryer (SunilEyela, Seongnam, Korea). The freeze-dried powder was dissolved in distilled water at a concentration of 1% (*w*/*v*).

### 2.4. Determination of Oligosaccharide Structure

#### 2.4.1. Thin-layer Chromatography

TLC was performed as described in a previous study [17]. Briefly, TLC was performed using silica gel 60F_254_ (Merck, Darmstadt, Germany). Samples were spotted onto a TLC plate and developed twice in a presaturated chromatographic chamber using a developing solvent (nitromethane (Sigma-Aldrich, St. Louis, MO, USA):*n*-propyl alcohol (Samchun, Gyeonggi, Korea):distilled water, 2:5:1.5, *v*/*v*/*v*). The developed TLC plate was then dipped in 0.3 g/L (*w*/*v*) *N*-(1-naphthyl) ethylenediamine dihydrochloride (Sigma-Aldrich) and 5 g/L (*v*/*v*) sulfuric acid (Duksan, Seoul, Korea) in methanol (CARLO ERBA Reagents S.A.A., Val de Reuil, France) and then baked at 121 °C for 5 min. Glucose polymer (G1–G8; glucose, maltose, maltotriose, maltotetraose, maltopentaose, maltohexaose, maltoheptaose, and maltooctaose, Carbosynth Co., Berkshire, UK) was used as a standard sugar.

#### 2.4.2. HPAEC-PAD Analysis

The purified oligosaccharides produced by *Leu. lactis* SBC001 were analyzed by high-performance anion-exchange chromatography with pulsed amperometric detection (HPAEC-PAD) (DX 500 Chromatography System, Dionex, Sunnyvale, CA, USA). The sample injection volume was 50 μL, and the oligosaccharides were analyzed using a CarboPac PA–1 column (4 × 250 mm, Dionex) and a CarboPac PA–1 guard column (4 × 50 mm, Dionex). The mobile phase was 150 mM sodium hydroxide for the first 15 min and 600 mM sodium acetate (in 150 mM sodium hydroxide) for the next 5 min, followed by 150 mM sodium hydroxide for the final 10 min at a flow rate of 1.0 mL/min.

#### 2.4.3. Size exclusion HPLC Analysis

The molecular weight of the oligosaccharides was determined using high-performance liquid chromatography (HPLC) (UltiMate^TM^ 3000 RSLCnano system, Thermo Fisher Scientific, Inc., Waltham, MA, USA), with an OHpak SB-802.5 column (8.0 × 300 mm, Shodex, New York, NY, USA). The oligosaccharides were dissolved in distilled water at a concentration of 100 mg/mL, and 100 µL of sample was injected for analysis and detected using an refractive index (RI) (RI-101, Shodex). The column mobile phase was distilled water, the flow rate was 0.4 mL/min, and the column oven temperature was 35 °C. Glucose polymer (DP 1–8, Carbosynth Co.) was used as a standard sugar.

#### 2.4.4. Analysis of Monosaccharide Composition

The oligosaccharides were hydrolyzed by heating at 121 °C for 2 h in 4 M trifluoroacetic acid (TFA). TFA was then removed by N_2_ gas flow, and the monosaccharide composition of the oligosaccharides was analyzed by HPAEC-PAD and TLC.

#### 2.4.5. Analysis of the Composition of Glycosidic Bonds by Enzymatic Analysis

Various carbohydrate-hydrolyzing enzymes were applied to the oligosaccharides for analyzing the composition of the glycosidic bonds. The oligosaccharides (200 mg/mL, dissolved in distilled water) were treated with 10 mU of α-amylase (Cat. # A9857, Sigma-Aldrich), 100 mU of α-glucosidase (Cat. # G3651, Sigma-Aldrich), 520 mU of amyloglucosidase (Cat. # A7420, Sigma-Aldrich), 1.4 U of pullulanase M1 (Cat. # E-PULKP, Megazyme, Chicago, IL, USA), 100 mU of β-glucosidase (Cat. # G0395, Sigma-Aldrich), and 10 mU of β-1,3-d-glucanase (Cat. # 67138, Sigma-Aldrich). The reaction mixtures were incubated at 37 °C for 1 h, and the products were determined by TLC.

#### 2.4.6. Analysis of Linkage Ratio by ^1^H-NMR Spectroscopy

The relative abundance of α-1,4 and α-1,6 linkages in the oligosaccharides was determined by 400 MHz ^1^H-NMR spectroscopy (JeolJNM-LA400 with LFG, JEOL, Tokyo, JAPAN) and Delta NMR Processing and Control Software version 5.3 (JEOL USA, Inc., Peabody, MA, USA). The oligosaccharides (10 mg/mL) were dissolved in deuterium oxide (D_2_O) and freeze-dried. The oligosaccharides were then dissolved in D_2_O (10 mg/mL) and analyzed by ^1^H-NMR operated at 80 °C.

### 2.5. Prebiotic Effects of Purified Oligosaccharides

#### 2.5.1. Bacterial and Yeast Strains and Culture Conditions

A total of 24 bacterial and yeast strains were obtained from Korean Culture Center of Microorganisms (KCCM, Seoul, Korea), including *Bifidobacterium adolescentis* KCCM 11206, *B. animalis* KCCM 11209, *B. bifidum* KCCM 12096, *B. breve* KCCM 42255, *B. longum* KCCM 11953, *Enterococcus faecium* KCCM 12118, *Lactobacillus acidophilus* KCCM 32820, *L. casei* KCCM 12452, *L. fermentum* KCCM 35469, *L. helveticus* KCCM 40989, *L. johnsonii* KCCM 41274, *L. paracasei* KCCM 40995, *L. pentosus* KCCM 40997, *L. plantarum* KCCM 12116, *L. rhamnosus* KCCM 32405, *L. salivarius* KCCM 40210, *Lactococcus lactis* KCCM 40104, *Leuconostoc citreum* KCCM 12030, *Leu. mesenteroides* KCCM 11325, *Pediococcus pentosaceus* KCCM 11902, *Streptococcus thermophilus* KCCM 35496, and *Weissella cibaria* KCCM 41287. These strains were cultured in MRS medium in the presence or absence of sugar or prebiotics at 37 °C for 48 h. All lactic acid bacteria were cultured using a capped tube without agitation, and all *Bifidobacterium* strains were cultured in anaerobic condition using the GasPak^TM^ system (BD). *S. cerevisiae* KCCM 50549 and *Zygosaccharomyces rouxii* KCCM 12066 were also obtained from KCCM and cultured using yeast extract malt extract (YM) medium (BD), which consisted of 10 g/L glucose, 3 g/L malt extract, 5 g/L peptone, and 3 g/L yeast extract, at 30 °C for 48 h.

#### 2.5.2. Media for Bacterial and Yeast Strains for the Determination of Prebiotic Effect

To determine the prebiotic effect of oligosaccharides, bacterial and yeast strains were incubated with medium in the presence or absence of sugar or oligosaccharides as a sole carbon source. Modified MRS (m-MRS) and modified YM (m-YM), which had no carbon sources, were prepared as described in a previous study [17]. For LAB, m-MRS was used as a negative control, and m-MRS containing 1% (*w*/*v*) dextrose was used as a positive control. For yeast strains, modified YM (m-YM) was used as a negative control, and YM containing 1% (*w*/*v*) dextrose was used as a positive control. Oligosaccharides were added to the liquid growth medium at a concentration of 1% (*w*/*v*). FOSs (from chicory, Cat. # F8052, Sigma-Aldrich) were also added to the growth medium at a concentration of 1% (*w*/*v*) as a reference.

#### 2.5.3. Determination of Viable Cell Number of Bacterial and Yeast Strains

A colony of LAB and *Bifidobacterium* sp. and a colony of yeast were seeded in MRS broth and YM broth, respectively, and incubated at 37 °C and at 30 °C at 250 rpm, respectively, for 24 h. Culture broth (1% (*v*/*v*)) was inoculated into the above-described media (described in Section 2.5.2). The culture broth was sampled at 0, 6, 12, 24, and 48 h. *Bifidobacterium* sp. and LAB were spread onto an MRS agar plate, and yeast cells were spread onto a YM agar plate. After the plates were incubated at 37 °C for 24 h, the viable cell number was calculated by counting the number of colonies on each plate and expressed as log CFU/mL.

### 2.6. Anti-inflammatory Activity of Oligosaccharides

#### 2.6.1. Cell Culture

RAW 264.7 cells, murine macrophages, were purchased from the Korean Cell Line Bank (Seoul, Korea). These cells were cultured in Dulbecco’s Modified Eagle Medium (DMEM; Gibco, Grand Island, NY, USA) supplemented with 10% bovine serum (Gibco, Grand Island, NY, USA) and 1% penicillin-streptomycin (Gibco) at 37 °C in a 5% CO_2_ incubator (Thermo Fisher Scientific, Waltham, MA, USA).

#### 2.6.2. Cytotoxicity on RAW 264.7 Cells

RAW 264.7 cells were plated in a 96-well plate (2 × 10^4^ cells/well) and treated with various concentrations (3.9, 7.8, 15.6, 31.2, 62.5, 125, 250, 500, 1000, and 10,000 μg/mL) of oligosaccharides for 24 h. The Cell Counting Kit 8 (CCK-8) reagent (DOJINDO Laboratories, Kumamoto, Japan) was added, and RAW 264.7 cells were incubated for 2 h. The viability of RAW 264.7 cells was evaluated by measuring the absorbance at 450 nm using a microplate reader (Epoch, Biotek Instruments, Inc., Winooski, VT, USA).

#### 2.6.3. Measurement of Nitric Oxide Production

The nitric oxide (NO) production by RAW 264.7 cells was measured using the Griess reagent. RAW 264.7 cells were plated in a 24-well plate (2 × 10^5^ cells/well) and pretreated with various concentrations (3.9, 7.8, 15.6, 31.2, 62.5, 125, 250, 500, 1000, 10,000 μg/mL) of oligosaccharides or 1 mg/mL FOS for 1 h. Then, cells were treated with 1 μg/mL lipopolysaccharides (LPS; from *Escherichia coli* K-235, Cat. # L2018, Sigma-Aldrich) for 24 h. Next, an appropriate amount of sample was mixed with 100 μL of Griess reagent (1% sulfanilamide, 0.1% *N*-(1-naphthyl)-ethylenediamine dihydrochloride, and 2.5% phosphoric acid) and allowed to react at room temperature for 20 min. The produced NO was measured using a microplate reader (Epoch, Biotek Instruments, Inc., Winooski, VT, USA) at a wavelength of 450 nm.

#### 2.6.4. cDNA Synthesis and Real-time PCR

Real-time PCR was used to measure the expression levels of inducible nitric oxide synthase (iNOS) gene, cytokine genes, including interleukin (IL)-1β and IL-6, and tumor necrosis factor (TNF)-α gene, using glyceraldehyde 3-phosphate dehydrogenase (GAPDH) gene as the housekeeping gene. After treating RAW 264.7 cells, which were cultured in a 6-well plate with oligosaccharides for 1 h, RAW 264.7 cells were treated with LPS (1 µg/mL) for 24 h and RNA was isolated using the easy-BLUE^TM^ Total RNA Extraction kit (iNtRON Biotechnology, Inc., Seongnam, Korea) according to the manufacturer’s instructions. Subsequently, cDNA was synthesized using the Transcriptor First Strand cDNA Synthesis Kit (Roche, Basel, Switzerland). Real-time PCR was conducted according to the manufacturer’s instructions (LightCycler96, Roche, Basel, Switzerland) using the FastStart Essential DNA Green Master Kit (Roche, Basel, Switzerland). The primer sequences used for the analysis of mRNA expression are shown in Table 1. The expression levels of cytokine mRNA relative to those of GAPDH mRNA were analyzed using the 2^−ddCT^ method.

#### 2.6.5. Western Blotting

RAW 264.7 cells were plated in a 6-well plate (1 × 10^6^ cells/well), pretreated with various concentrations (10, 100, and 1000 μg/mL) of oligosaccharides for 1 h, and then treated with 1 μg/mL LPS as a control for 24 h. After treatments, the cells were washed with phosphate-buffered saline (PBS) and lysed with radioimmunoprecipitation assay (RIPA) cell lysis buffer (Cell Signaling Technology, Beverly, MA, USA). The supernatant was collected after centrifugation at 14,000× *g* for 10 min at 4 °C. The protein content was measured using the BCA protein assay kit (Thermo Scientific, Waltham, MA, USA). Proteins (50 μg) for each sample were separated by 10% or 12% sodium dodecyl sulfate-polyacrylamide gel electrophoresis (SDS-PAGE) and transferred onto nitrocellulose membranes (Bio-Rad Laboratories, Inc.). The membranes were blocked with 5% skim milk at room temperature for 2 h and then incubated overnight with primary antibodies (1:1000; Cell Signalling Technology, Danvers, MA, USA) at 4 °C. After washing three times with Tris-buffered saline containing 0.5% Tween 20 (TBST), the membranes were treated with the corresponding secondary antibody (1:3000; anti-rabbit IgG, Cell Signalling Technology) at 37 °C for 2 h. The membranes were again washed three times with TBST, after which the chemiluminescent signals emitted after incubation with secondary antibodies were detected with EzWestLumi, plus chemiluminescent substrate (ATTO Corporation, Tokyo, Japan), using Odyssey LCI Image software (LI-COR Biosciences, Lincoln, NE, USA).

### 2.7. Statistical Analysis

Data were expressed as mean ± standard deviation (SD) from triplicate experiments. Statistical analyses were conducted using SPSS 23 (SPSS Inc., Chicago, IL, USA). Statistical significance between groups was determined by a one-way analysis of variance (ANOVA), followed by Duncan’s multiple range test (*p* < 0.05).

## 3. Results and Discussion

### 3.1. Optimization of Oligosaccharide Production

*Leu. lactis* SBC001 was isolated from chives in our laboratory and selected as an oligosaccharide-producing strain by detecting the formation of slimy colonies on sucrose-containing agar medium and by measuring the glucansucrase activity [23].

To optimize the conditions for oligosaccharide production by *Leu. lactis* SBC001, RSM was used with a central composite design. From the preliminary experiments, the initial pH, sucrose concentration, and culture temperature were chosen as factors for optimization. As shown in Table 2, when the initial pH (X_1_) was 7.0, sucrose concentration (X_2_) was 0.4 M and culture temperature (X_3_) was 29 °C, the maximum SBC-oligosaccharide production was obtained, with 209.44 as a relative peak area. The mathematical regression equation was given as follows:

Y = 54.82 + 10.86X_1_ + 7.46X_2_ − 47.54X_3_ − 3.31X_1_^2^ − 0.31X_2_^2^ + 22.06X_3_^2^ + 4.23X_1_X_2_ − 20.57X_1_X_3_ − 8.31X_2_X_3_ (1)

where Y denotes oligosaccharide production, X_1_ is the initial pH, X_2_ is the sucrose concentration, and X_3_ indicates the culture temperature.

The appropriateness of the experimental model used in this study was confirmed assuming that a significance of 0.000 was suitable for the model, and in the lack-of-fit test, the significance was 0.000 (Table 3).

**Figure 1:**
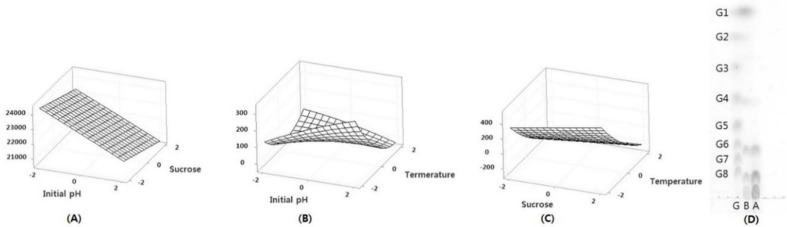
Three-dimensional response surface plots (**A**–**C**) and thin-layer chromatography (TLC) (**D**) showing the optimization of oligosaccharide production. (**A**) Response surface plot showing the effect of initial pH (X1) and sucrose concentrations (X2). (**B**) Response surface plot showing the effect of initial pH (X1) and fermentation temperature (X3). (**C**) Response surface plot showing the effect of sucrose concentrations (X2) and fermentation temperature (X3). (**D**) TLC chromatogram of oligosaccharides before and after purification (G, glucose polymers G1–G8; B, before purification; A, after purification by Bio-gel P2).

Figure 1 shows the three-dimensional plot for the effect of each variable on the production of oligosaccharides from *Leu. lactis* SBC001. Using these optimum conditions, oligosaccharides were produced by *Leu. lactis* SBC001 and purified by Bio-gel P2 size exclusion column chromatography. The resulting purified oligosaccharides had a DP of 4 to 9, as shown in Figure 1D. A previous study demonstrated that the maximum production of gluco-oligosaccharides from *Leu. lactis* CCK940 could be obtained using a culture temperature of 30 °C and sucrose and maltose concentrations of 0.28 and 0.22 M, respectively, as determined by the RSM study [13].

### 3.2. Structural Analysis of the Oligosaccharides Produced by Leu. lactis SBC001

#### 3.2.1. Analysis of the Monosaccharide Composition of Oligosaccharides

**Figure 2:**
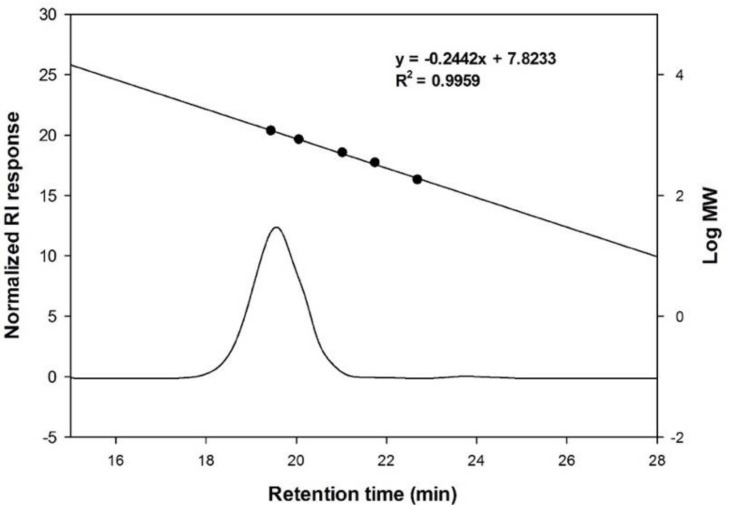
Size exclusion high-performance liquid chromatography (HPLC) of the oligosaccharides.

The molecular weight of the purified oligosaccharides was measured by size exclusion chromatography. Among the oligosaccharides, the molecular weight of the oligosaccharide with the highest concentration was determined as 1137 Da, which corresponded to DP 7 of glucose unit (Figure 2). In the previous study on gluco-oligosaccharides produced by *Leu. lactis* CCK940, the molecular weight of the oligosaccharide with the highest concentration was 942 Da, which was smaller than that of the oligosaccharides produced by *Leu. lactis* SBC001 [17]. It has also been reported that the molecular weight range of chitosan oligosaccharides was 1000–1600 Da [24], and that of pectin oligosaccharides was 1000–3000 Da [25].

**Figure 3:**
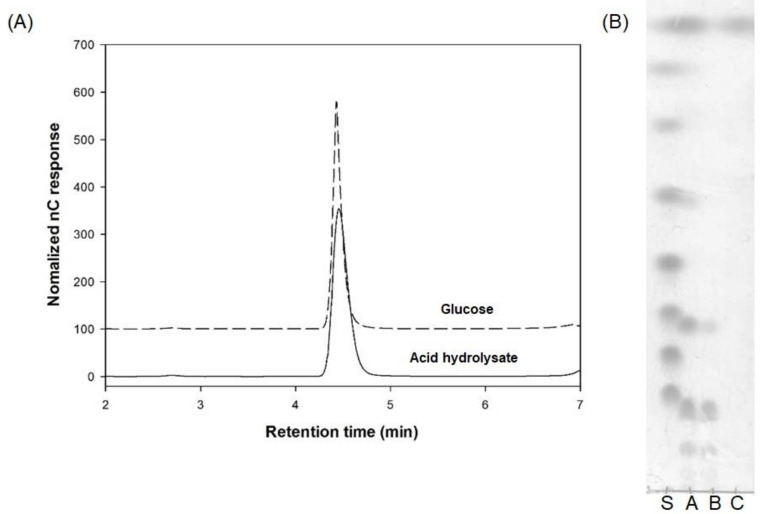
Carbohydrate composition of the oligosaccharides. (**A**) high-performance anion-exchange chromatography with pulsed amperometric detection (HPAEC-PAD) chromatogram of 0.1 mg glucose in 10 mL distilled water and 1 mg acid hydrolysate in 10 mL distilled water. (**B**) TLC chromatogram of the oligosaccharides before and after purification of SBC-oligosaccharides and acid hydrolysate (S, standard; A, oligosaccharides before purification; B, oligosaccharides after purification; C, acid hydrolysates of purified oligosaccharides).

As shown in Figure 3, the hydrolysates consisted of glucose, which indicated that the oligosaccharides produced by *Leu. lactis* SBC001 were gluco-oligosaccharides that consisted of only glucose. The oligosaccharides produced by *Leu. lactis* CCK940 were also gluco-oligosaccharides [17].

#### 3.2.2. Enzymatic Hydrolysis of SBC-Oligosaccharides

**Figure 4:**
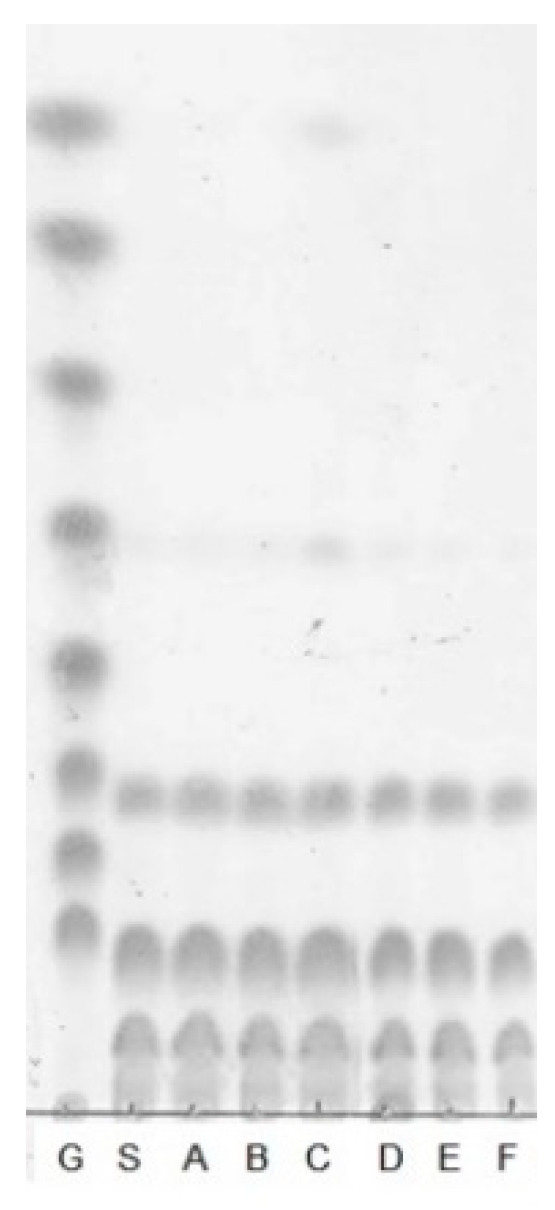
Enzymatic hydrolysis of SBC-oligosaccharides. G, standard; S, oligosaccharides produced by *Leu. lactis* SBC001; A, oligosaccharides treated with α-amylase; B, oligosaccharides treated with α-glucosidase; C, oligosaccharides treated with amyloglucosidase; D, oligosaccharides treated with pullulanase M1; E, oligosaccharides treated with β-glucosidase; F, oligosaccharides treated with β-1,3-d-glucanase.

The oligosaccharides were hydrolyzed with various carbohydrate-hydrolyzing enzymes, and their products were analyzed to determine the types of glycosidic bonds in the oligosaccharides. As shown in Figure 4, the purified oligosaccharides were hydrolyzed by amyloglucosidase but not by α-amylase, α-glucosidase, β-glucosidase, or β-1,3-d-glucanase. Pullulanase M1 slightly hydrolyzed the oligosaccharides.

α-Amylase (EC 3.2.1.1) is one of the human digestive enzymes that hydrolyzes α-1,4 glycosidic linkages in starch, amylopectin, amylose, and glycogen [26]. Similarly, α-glucosidase (EC 3.2.1.20) is an intestinal cell surface membrane enzyme playing a role in the hydrolysis of α-1,4 glycosidic linkages [27]. β-Glucosidase (EC 3.2.1.21) hydrolyzes the β-1,4-glycosidic linkages in oligosaccharides, amino-, or alkyl-β-d-glucosides at non-reducing ends, releasing β-d-glucose monomers [28]. β-1,3-d-glucanase (EC 3.2.1.6) catalyzes the hydrolysis of β-1,3 glycosidic linkages [29]. Amyloglucosidase (EC 3.2.1.3), also known as 1,4-α-d-glucan hydrolase, hydrolyzes both α-1,6 and α-1,4 glycosidic linkages in the oligosaccharides. It also catalyzes the cleavage of glucose units from the non-reducing ends of oligosaccharides and polysaccharides [30,31]. Pullulanase (EC 3.2.1.41) can cleave α-1,6 glycosidic linkages in amylopectin and pullulan, but it does not hydrolyze α-1,4 glycosidic linkages [32]. Based on these results, it was predicted that the oligosaccharides obtained from *Leu. lactis* SBC001 have α-1,6 and α-1,4 glycosidic linkages.

#### 3.2.3. ^1^H-NMR Analysis

^1^H-NMR spectroscopy is a useful method for the determination of linkage type, DP, and structural analysis of polysaccharides and oligosaccharides [33,34]. When the linkage ratio of the oligosaccharides produced by *Leu. lactis* SBC001 was analyzed by ^1^H-NMR spectroscopy, the chemical shifts of oligosaccharides, maltose, and waxy corn starch (WCS) were at 5.2–6.0 ppm, which are characteristic of the α-linkages (Figure S1). The results demonstrated that the oligosaccharides produced by *Leu. lactis* SBC001 consisted of 23.63% α-1,4 linkages and 76.37% α-1,6 linkages (Table 4).

Maltose and WCS, used for standard molecules, consist of α-1,4 glycosidic linkages and α-1,6 and α-1,4 glycosidic linkages, respectively. The oligosaccharides produced by *Leu. lactis* SBC001 were different from the other gluco-oligosaccharides or malto-oligosaccharides, wherein malto-oligosaccharides produced from *Lactobacillus reuteri* E81 consisted of α-1,6 and α-1,4 glycosidic linkages with ratios of 54% and 46%, respectively [35]. Lee et al. [17] reported that the linkages of gluco-oligosaccharides produced by *Leu. lactis* CCK940 comprised 22.4% α-1,4 linkages and 77.6% α-1,6 linkages, similar to the results of the present study.

### 3.3. Prebiotic Effect of Oligosaccharides on the Growth of Bacterial and Yeast Strains

**Figure 5:**
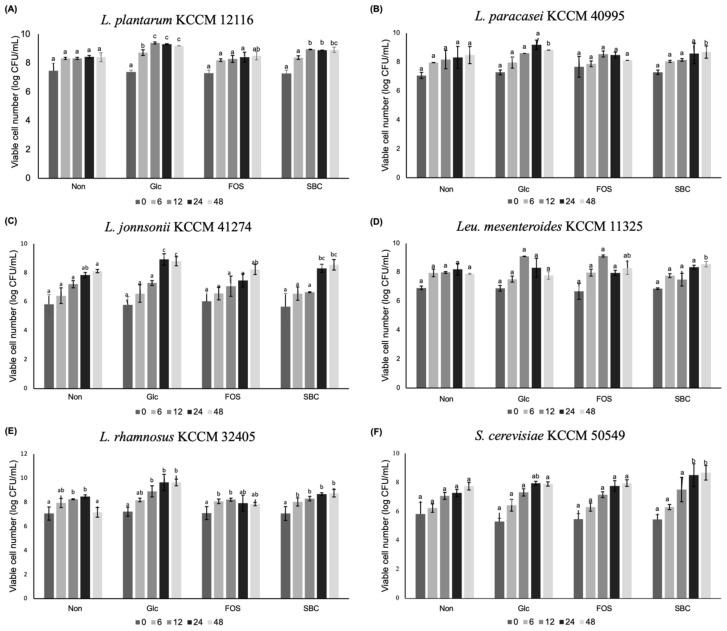
Prebiotic effects of the oligosaccharides produced by *Leu. lactis* SBC001 on bacterial and yeast strains. (**A**) *L. plantarum* KCCM 12116; (**B**) *L. paracasei* KCCM 40995; (**C**) *L. johnsonii* KCCM 41274; (**D**) *Leu. mesenteroides* KCCM 11325; (**E**) *L. rhamnosus* KCCM 32405; (**F**) *S. cerevisiae* KCCM 50549. Non, glucose-free medium; Glc, 1%(*w*/*v*) glucose-containing medium; FOS, glucose-free medium supplemented with 1%(*w*/*v*) FOS; SBC, glucose-free medium supplemented with 1%(*w*/*v*) oligosaccharides produced by *Leu. lactis* SBC001. Different letters among groups represent statistically significant differences (*p* < 0.05). 0, 6, 12, 24, and 48, mean incubation time (h) of bacterial and yeast cells.

To determine the prebiotic effect of oligosaccharides produced by *Leu. lactis* SBC001, 24 representative bacterial and yeast strains were selected and their growth was determined using the modified culture media supplemented with different carbon sources. Among the 24 strains, six strains, including *L. plantarum*, *L. paracasei*, *L. johnsonii*, *Leu. mesenteroides*, *L. rhamnosus*, and *S. cerevisiae*, showed higher viable cell numbers in glucose-free MRS supplemented with oligosaccharides at 48 h of incubation when compared with FOS that was used as a reference (Figure 5). For example, the viable cell numbers of *L. plantarum* were 8.90 ± 0.18 and 8.40 ± 0.28 log CFU/mL in oligosaccharide medium and FOS medium, respectively, at 48 h (Figure 5A). The viable cell numbers of strains were significantly increased by the supplementation of oligosaccharides compared with glucose-free MRS or glucose-free YM during 48 h of incubation. In the case of *S. cerevisiae*, the viable cell numbers were 8.09 ± 0.07 and 8.47 ± 0.33 log CFU/mL in oligosaccharide medium and FOS medium, respectively, at 48 h, which showed the prebiotic effect of oligosaccharides on yeast cells (Figure 5F).

The results demonstrated that the oligosaccharides produced by *Leu. lactis* SBC001 exhibited a higher prebiotic effect on some strains than FOSs that are widely used as prebiotics. In the previous study, the gluco-oligosaccharides produced by *Leu. lactis* CCK940 exhibited prebiotic effects on *Lactobacillus casei*, *L. pentosus*, *L. plantarum*, *W. cibaria*, *B. animalis*, and *S. cerevisiae*, which was different from this study result [17]. Several other studies have shown that each prebiotic oligosaccharide has its own prebiotic effect on specific probiotic strains [36,37,38]. The results of these studies indicate that the prebiotic effects of lactic acid strains vary depending on the type and structure of the oligosaccharides produced.

### 3.4. Anti-Inflammatory Effect of Oligosaccharides

#### 3.4.1. Effect of Oligosaccharides on RAW 264.7 Cell Viability and NO Production

Because some oligosaccharides exhibit anti-inflammatory activity on macrophages, we explored the anti-inflammatory effect of the oligosaccharides produced by *Leu. lactis* SBC001 on RAW 264.7 macrophage cells.

**Figure 6:**
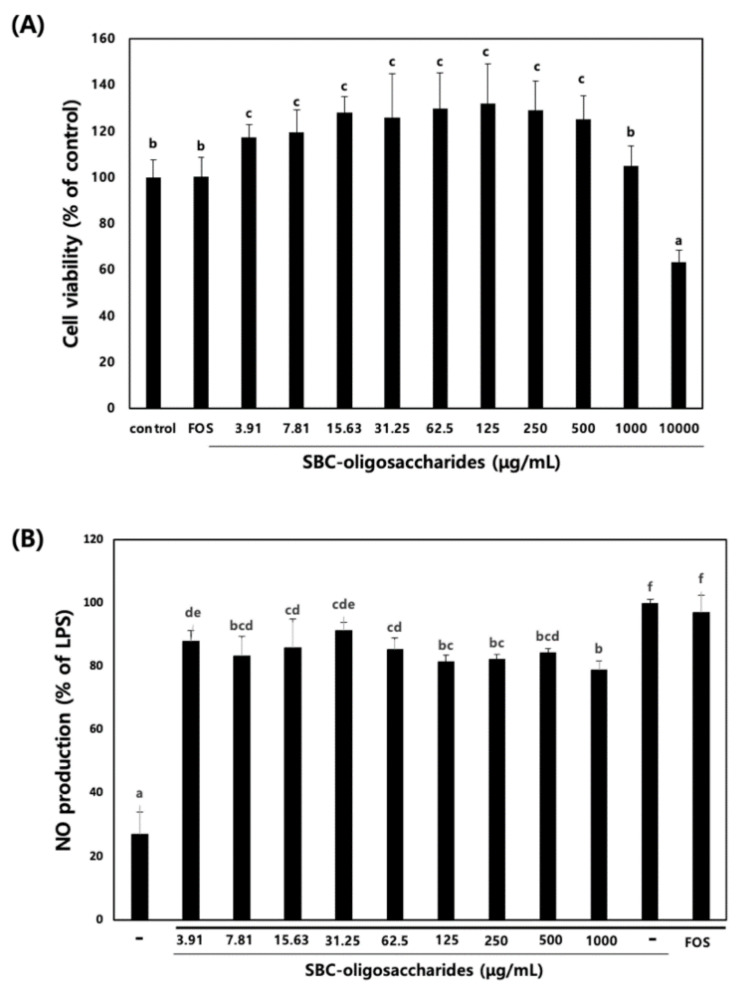
Effects of oligosaccharides on the cell viability and NO production in RAW 264.7 cells. (**A**) Effects of oligosaccharides on RAW 264.7 cell viability. (**B**) Effects of oligosaccharides on NO production in lipopolysaccharide (LPS)-stimulated RAW 264.7 cells. RAW 264.7 cells were treated with various concentrations of oligosaccharides for 1 h and then treated with 1 μg/mL LPS for 24 h. NO production was evaluated using the Griess reaction assay. Different letters show significant differences among groups at *p* < 0.05 (n ≥ 3).

The viability of RAW 264.7 cells was analyzed in the presence of different concentrations of purified oligosaccharides. When RAW 264.7 cells were treated with the oligosaccharides, the oligosaccharides did not exert cytotoxic effects on RAW 264.7 cells up to a concentration of 1000 μg /mL (Figure 6). Therefore, oligosaccharide concentrations of 10, 100, and 1000 μg/mL were used for further experiments. NO is a free radical molecule synthesized from l-arginine by NO synthase; the production of NO promotes the inflammatory response, and NO inhibitors can prevent the inflammatory response [39]. NO is not stable, and in the presence of water and oxygen, it converted into nitrite and nitrate, which can be detected using Griess reagent. When the cells were treated with LPS alone, NO production was stimulated, and its relative amount was set to 100%. The production of NO was decreased by 20% upon treatment with oligosaccharides at a concentration of 1000 µg/mL, whereas treatment with 1 mg/mL FOS did not decrease the NO production (Figure 6). This result indicated that the oligosaccharides produced by *Leu. lactis* SBC001 exhibited a higher anti-inflammatory activity than FOSs. FOSs are oligosaccharides which consist of fructose residues with a glucose residue at the end of the chain linked by beta-(2,1) glycosidic bonds, the structure of which is quite different from oligosaccharides produced by *Leu. lactis* SBC001. Even though their structural properties are different, FOSs also have anti-inflammatory activity similar to oligosaccharides produced by *Leu. lactis* SBC001 [40,41]

#### 3.4.2. Effect of Oligosaccharides on Cytokine Production

Various mediators of the inflammatory response, including iNOS, cyclooxygenase (COX)-2, chemokines, and proinflammatory cytokines (TNF-α, IL-1β, IL-6, and IL-10) are expressed on activated macrophages [42].

The activation of iNOS, inducible NO synthase, has been demonstrated to contribute to septic shock [43]. iNOS inhibitors regulate as therapeutic agents to influence gastrointestinal diseases and arthritis [44]. IL-6 acts as a multifunctional cytokine that increases phagocytosis and complements production and is defined as a B cell differentiation factor [45]. TNF-α, an important mediator cytokine, is involved in host defense against pathogens [46]. TNF-α induces apoptosis and is involved in the development of humoral immune response. IL-1β is released by macrophages and plays a role in the pathophysiology of rheumatoid arthritis [47].

**Figure 7:**
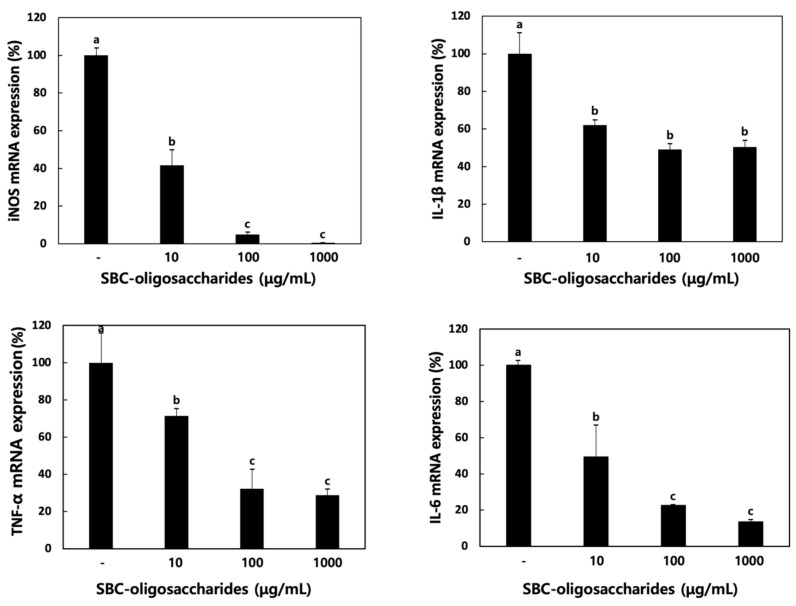
Relative mRNA expression of inducible NO synthase (iNOS) and inflammatory cytokines upon treatment with oligosaccharides in RAW 264.7 cells. RAW 264.7 cells were pretreated with oligosaccharides for 1 h and then stimulated with 1 μg/mL LPS. One-way ANOVA was used to compare group mean values, followed by Duncan’s *t*-test. Different letters represent significant differences among groups at *p* < 0.05 (n ≥ 3).

In the present study, the expression of iNOS and other proinflammatory cytokines was evaluated in RAW 264.7 cells treated with the oligosaccharides produced by *Leu. lactis* SBC001. As depicted in Figure 7, the mRNA expression levels of TNF-α, IL-1β, and IL-6 decreased in a dose-dependent manner after treatment with the oligosaccharides. Moreover, the iNOS mRNA expression levels were reduced after oligosaccharide treatment. These results indicated that the anti-inflammatory activity of the oligosaccharides was due to the repression of iNOS and other proinflammatory cytokines; however, the underlying mechanism needs to be elucidated in a further study.

#### 3.4.3. Effect of Oligosaccharides on the MAPK Signaling Pathway of RAW 264.7 Cells

Nuclear factor-kappa B (NF-κB) is an important target transcription factor in the inflammatory response [48]. The NF-κB signaling pathway plays an important role in the LPS-stimulated expression of inflammatory-associated genes and cytokines and is associated with toll-like receptor 4 (TLR4) that activates the downstream signaling pathways [49]. The inhibitor of κB (IκB) that is maintained in the cytoplasm regulates the activation of NF-κB. When LPS stimulates the degradation of IκB, phospho-IκB (p-IκB) results in the nuclear translocation of NF-κB. NF-κB in the nucleus stimulates the transcription of proinflammatory genes such as iNOS, TNF-α, IL-1β, and IL-6 [50].

**Figure 8:**
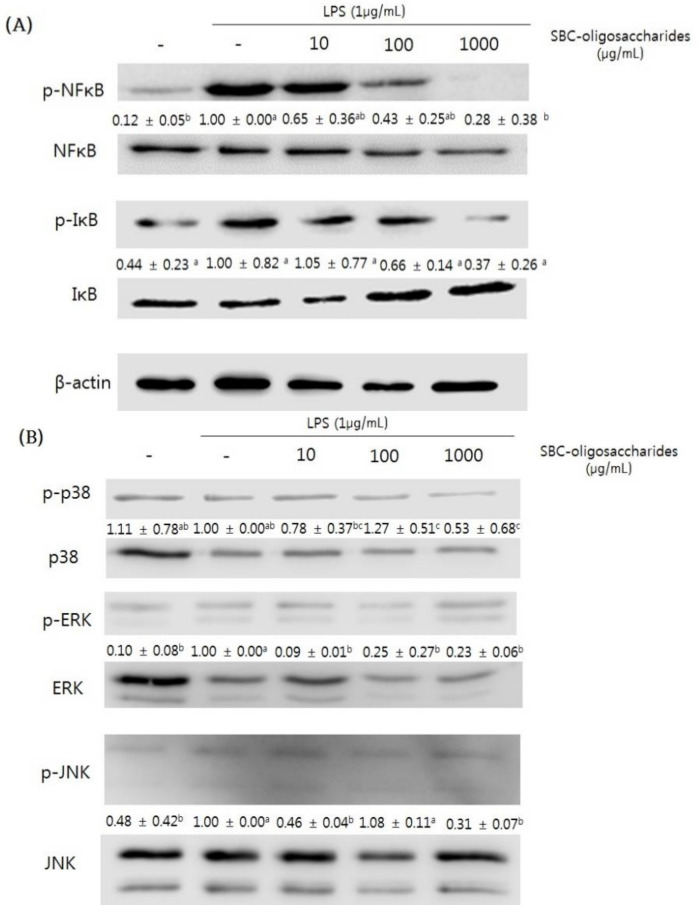
Effects of the oligosaccharides produced by *Leu. lactis* SBC001 on the nuclear factor-kappa B (NF-κB) signaling pathway and MAPK signaling pathways in RAW 264.7 cells. RAW 264.7 cells were pretreated with oligosaccharides for 1 h and then treated with 1 μg/mL LPS for 24 h. (**A**) Western blot images for the expression of genes in the NF-κB signaling pathway. (**B**) Western blot images for the expression of genes in the MAPK signaling pathways. One-way ANOVA was used for the comparison of group mean values, followed by Duncan’s multiple range test for assessing the significance of individual comparisons (*p* < 0.05) (n ≥ 3). Different letters represent significant differences among groups. The numbers between blots indicate the relative expression level.

In the present study, RAW 264.7 cells were treated with the oligosaccharides produced by *Leu. lactis* SBC001, and the expression of NF-κB and IκB was evaluated (Figure 8A). Treatment with oligosaccharides suppressed the expression of NF-κB and IκB in a dose-dependent manner.

The mitogen-activated protein kinase (MAPK) signaling pathways include extracellular signal-regulated kinases 1 and 2 (ERK1/ERK2), c-Jun N-terminal kinases (JNKs), and p38 MAPK [46]. The MAPK signaling pathways are associated with cell growth, differentiation, and death through regulation of the expression of numerous genes, and the LPS-stimulated expression of inflammatory mediators, including iNOS, COX-2, and inflammatory cytokines, is involved in the MAPK signaling pathways [51]. In addition, inhibition of MAPK phosphorylation can reduce inflammatory diseases. As shown in Figure 8B, when LPS-stimulated RAW 264.7 cells were pre-treated with the oligosaccharides, the expression of phosphorylated p38, ERK, and JNK was decreased. Based on these results, it is predicted that treatment of RAW 264.7 cells with the oligosaccharides reduces the inflammatory response by suppressing the inflammatory-associated genes and cytokines in the NF-κB signaling pathway and MAPK signaling pathways.

Numerous studies have reported about the anti-inflammatory activities of oligosaccharides. Yoon et al. demonstrated that chitosan-oligosaccharides (COS) suppressed the LPS-induced secretion of TNF-α and IL-6 as well as NO in the medium, and this anti-inflammatory effect of COS was achieved by the modulation of the TNF-α pathway [52]. Pangestuti et al. reported about the effects of COS on the modulation of inflammatory mediators in LPS-stimulated BV microglial cells. In their study, it was shown that COS suppressed the production of NO and prostaglandin E2 by inhibiting the expression of iNOS and COX-2, and this effect was due to the suppression of the phosphorylation of JNK and p38 MAPK [53]. Chung also reported that *Eubacterium eligens*, a species belonging to Firmicutes, utilizes apple pectin as a prebiotic source and promotes the production of the anti-inflammatory cytokine IL-10 in host cells [54]. Although some studies have explored the immunomodulatory activity of gluco-oligosaccharides, there has been no study on the anti-inflammatory activity of gluco-oligosaccharides [16].

## 4. Conclusions

There are several oligosaccharides that exhibit prebiotic effects on bacterial and yeast strains such as *Lactobacillus* sp., *Bifidobacterium* sp., and *S. cerevisiae*. There are also several oligosaccharides that exhibit anti-inflammatory or immunomodulatory activity. However, to our knowledge, there has been no report on the anti-inflammatory activity of gluco-oligosaccharides produced by *Leu. lactis*, and this is the first report on this aspect. FOSs are one of the most used prebiotic oligosaccharides worldwide, and it is meaningful that the prebiotic and anti-inflammatory activities of gluco-oligosaccharides produced by *Leu. lactis* SBC001 are higher than those of FOSs. This implies that gluco-oligosaccharides produced by *Leu. lactis* SBC001 could be used as a substitute for FOSs. It is predicted that their anti-inflammatory activity was due to the reduction in the inflammatory response by suppressing the inflammatory-associated genes and cytokines in the NF-κB signaling pathway and the MAPK signaling pathways.

## Figures and Tables

**Table 1:** Primer sequences for real-time PCR.

Gene	Primer Sequence	
iNOS	Forward Reverse	5′-AATGGCAACATCAGGTCGGCCATCACT-3 ′5′-GCTGTGTGTCACAGAAGTCTCGAACTC-3′
IL-6	Forward Reverse	5′-CAAGAGACTTCCATCCAGTTGC-3 ′5′-TTGCCGAGTTCTCAAAGTGAC-3′
IL-1β	Forward Reverse	5′-ATGGCAACTGTTCCTGAACTCAACT-3 ′5′-CAGGACAGGTATAGATTCTTTCCTT-3′
TNF-α	Forward Reverse	5′-ATGAGCACAGAAAGCATGATCCG-3 ′5′-CCAAAGTAGACCTGCCCGGACTC-3′
GAPDH	Forward Reverse	5′-ATCCCATCACCATCTTCCAG-3 ′5′-CCTGCTTCACCACCTTCTTG-3′

**Table 2:** Central composite design for three factors and the resulting SBC-oligosaccharide production.

Run	Factor Value			SBC-Oligosaccharide Production (Relative Peak Area)
	Initial pH	Sucrose (M)	Temperature (°C)	
1	5.0 (−1)	0.2 (−1)	29 (−1)	117.43 ± 2.71 ^g^
2	7.0 (1)	0.2 (−1)	29 (−1)	154.47 ± 5.60 ^i^
3	5.0 (−1)	0.4 (1)	29 (−1)	134.52 ± 2.92 ^h^
4	7.0 (1)	0.4 (1)	29 (−1)	209.44 ± 0.60 ^k^
5	5.0 (−1)	0.2 (−1)	37 (1)	28.98 ± 1.97 ^b^
6	7.0 (1)	0.2 (−1)	37 (1)	5.02 ± 0.67 ^a^
7	5.0 (1)	0.4 (1)	37 (1)	33.81 ± 0.15 ^bc^
8	4.0 (−2)	0.3 (0)	33 (0)	5.47 ± 2.09 ^a^
9	8.0 (2)	0.3 (0)	33 (0)	1.28 ± 0.34 ^a^
10	6.0(0)	0.1 (−2)	33 (0)	57.62 ± 2.66 ^e^
11	6.0 (0)	0.5 (2)	33 (0)	37.76 ± 8.23 ^bc^
12	6.0 (0)	0.3 (0)	41 (2)	53.50 ± 2.23 ^de^
13	6.0 (0)	0.3 (0)	25 (−2)	185.60 ± 1.14 ^j^
14	6.0 (0)	0.3 (0)	41 (2)	76.03 ± 0.25 ^f^
15	6.0 (0)	0.3 (0)	33 (0)	57.67 ± 21.04 ^e^
16	6.0 (0)	0.3 (0)	33 (0)	44.46 ± 2.84 ^cde^
17	6.0 (0)	0.3 (0)	33 (0)	53.30 ± 10.6 ^de^
18	6.0 (0)	0.3 (0)	33 (0)	56.38 ± 10.61 ^de^
19	6.0 (0)	0.3 (0)	33 (0)	42.88 ± 3.68 ^bcd^
20	6.0 (0)	0.3 (0)	33 (0)	49.78 ± 1.71 ^de^

Different superscripts represent significant differences among groups.

**Table 3:** Analysis of variance of the experimental results using a central composite design.

Variables	DF	Adj SS	Adj MS	F-Value	*p*-Value
Model	9	114,385	12,709.4	19.59	0.000
Linear	3	32,181	107,26.9	16.54	0.000
Quadratic	3	28,351	9450.3	14.57	0.000
Cross-product	3	8162	2720.7	4.19	0.014
X_1_	1	7033	7032.5	10.84	0.003
X_2_	1	827	826.8	1.27	0.268
X_3_	1	18,490	18,490.3	28.5	0.000
X_1_^2^	1	549	549.4	0.85	0.365
X_2_^2^	1	5	4.8	0.01	0.932
X_3_^2^	1	24,461	24,461	37.71	0.000
X_1_X_2_	1	286	285.9	0.44	0.512
X_1_X_3_	1	6772	6771.7	10.44	0.003
X_2_X_3_	1	1104	1104.5	1.7	0.202
Residual	50	19,461	648.7	-	-
Lack of fit	5	18,323	3664.6	80.5	0.000
Prue error	45	1138	45.5	-	-
Cor Total	59	133,846	-	-	-

**Table 4:** The proportion of glycosidic linkages in oligosaccharides and waxy corn starch (WCS).

	α-1,4 Glycosidic Linkages (%)	α-1,6 Glycosidic Linkages (%)	Ratio ofα-1,4 to α-1,6
Waxy corn starch	96.9 ± 0.11	3.11 ± 0.11	31.22 ± 1.10
Oligosaccharides produced by *Leu. lactis* SBC001	23.63 ± 0.91	76.37 ± 0.91	0.31 ± 0.01

## Data Availability

The data presented in this study are available on request from the corresponding author.

## Supplementary Materials

The following materials are available online at https://www.mdpi.com/2076-2607/9/1/200/s1, Figure S1: Comparison of the 1H-NMR spectra (400 MHz, D_2_O) for the oligosaccharides produced by *Leu. lactis* SBC001, maltose, and waxy corn starch (WCS). Table S1: Experimental design of optimization conditions for CCK-oligosaccharides. Table S2: Optimization condition experimental design of initial pH, sucrose concentration, culture temperature for oligosaccharide production from *Leu. lactis* SBC001.
