# Supplementary material for: Production Optimization, Structural Analysis, and Prebiotic- and Anti-Inflammatory Effects of Gluco-Oligosaccharides Produced by Leuconostoc lactis SBC001
Source: Microorganisms. 2021 Jan 19;9(1):200. doi: 10.3390/microorganisms9010200 (PMC7835818; doi:10.3390/microorganisms9010200)
Supplement: Supplementary file 1 [file microorganisms-09-00200-s001.pdf]

Table S1. Experimental design of optimization conditions for CCK–oligosaccharides.

Symbol	Factor	-2	-1	0	+1	+2
X_1	Initial pH	4.0	5.0	6.0	7.0	8.0
X_2	Sucrose (M)	0.1	0.2	0.3	0.4	0.5
X_3	Temperature(℃)	25	29	33	37	41

Table 2. Optimization condition experimental design of initial pH, sucrose concentration, culture temperature for oligosaccharides production from *Leu. lactis* SBC001.

Run	Factor value		
	Initial pH	Sucrose (M)	Temperature (℃)
1	7 (1)	0.4 (1)	37 (1)
2	6 (0)	0.3 (0)	33 (0)
3	6 (0)	0.5 (2)	33 (0)
4	7 (1)	0.4 (1)	37 (1)
5	6 (0))	0.3 (0)	41 (2)
6	7 (1)	0.2 (-1)	37 (1)
7	6 (0)	0.5 (2)	33 (0)
8	6 (0)	0.3 (0)	33 (0)
9	6 (0))	0.3 (0)	41 (2)
10	6 (0)	0.3 (0)	33 (0)
11	5 (-1)	0.2 (-1)	37 (1)
12	6 (1)	0.4 (1)	29 (-1)
13	8 (2)	0.3 (0)	33 (0)
14	7 (1)	0.4 (1)	29 (-1)
15	6 (0)	0.3 (0)	33 (0)
16	6 (0)	0.3 (0)	33 (0)
17	5 (-1)	0.2 (-1)	29 (-1)
18	6 (0)	0.3 (0)	33 (0)
19	6 (0)	0.3 (0)	25 (-2)
20	6 (0)	0.3 (0)	33 (0)

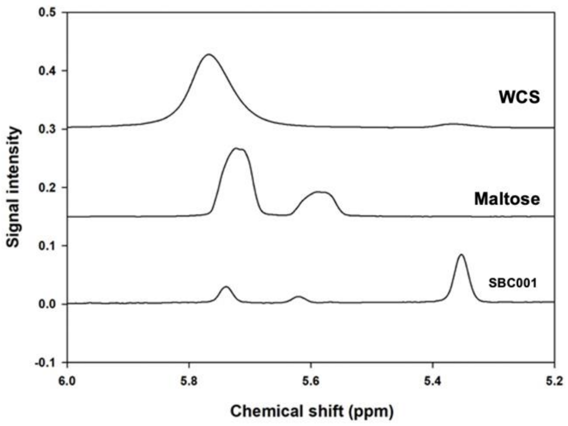

Figure S1. Comparison of the ^{1}H-NMR spectra (400 MHz, D_2O) for the oligosaccharides from Leu. lactis SBC001, maltose, and waxy corn starch (WCS).
